# Association of Osteoprotegerin Gene Polymorphisms (rs2073618 and rs3102735) with Bone Mineral Density in Childhood Acute Lymphoblastic Leukemia Survivors

**DOI:** 10.3390/children13101420

**Published:** 2026-10-16

**Authors:** Danijela Jovančić Petković, Jelena Bašić, Dragana Jugović, Vesna Bogićević, Sandra Stanković, Jelena Vojinović

**Affiliations:** 1 Medical Faculty, University of Niš, 18000 Niš, Serbia; jelena.basic@medfak.ni.ac.rs (J.B.); sandra.stankovic@medfak.ni.ac.rs (S.S.); jelena.vojinovic@medfak.ni.ac.rs (J.V.); 2 Clinic for Pediatrics, University Clinical Center Niš, 18000 Niš, Serbia; dragana.jugovic@live.com (D.J.); vajav@mts.rs (V.B.)

**Keywords:** lymphoblastic leukemia, survivors, bone mineral density, osteoprotegerin

## Abstract

**Highlights:**

**What are the main findings?**

- Low bone mineral density is a frequent late side effect in childhood acute lymphoblastic leukemia survivors.
- Besides treatment intensity, individual susceptibility could be an important risk factor for the development of this complication.

**What are the implications of the main findings?**

- Childhood cancer survivors need systematic long-term follow-up.
- Identification of survivors at risk for development of osteoporosis could be important for the early prevention of this late complication.

**Abstract:**

**Background**: Reduced bone mineral density (BMD) is a frequent late complication among survivors of childhood acute lymphoblastic leukemia (ALL). Osteoprotegerin is an important regulator of bone remodeling, and genetic variations within TNFRSF11B, which encodes osteoprotegerin, may contribute to individual susceptibility to impaired bone health. This study investigated the association between two polymorphisms (rs2073618 and rs3102735) and BMD in survivors of childhood ALL. **Methods**: This single-center cohort study included 60 survivors of childhood ALL. Lumbar spine BMD was assessed by dual-energy X-ray absorptiometry, and age-, sex-, and height-adjusted Z-scores were calculated. Low BMD was defined as a Z-score ≤ −1. Genotyping of rs2073618 and rs3102735 polymorphisms was performed using TaqMan real-time polymerase chain reaction assays. **Results**: Low BMD was observed in 22 patients (36.7%). We found no significant associations between BMD and demographic or treatment-related factors. Mean BMD Z-scores differed significantly among rs2073618 genotypes (ANOVA, *p* = 0.030). Carriers of the GG genotype had significantly higher BMD Z-scores than carriers of the CC or CG genotype (−0.05 ± 1.29 vs. −0.78 ± 0.88; *p* = 0.026). After adjustment for sex, treatment risk group, dexamethasone exposure, and age at diagnosis, rs2073618 remained independently associated with BMD Z-score (β = 0.337, *p* = 0.019). No significant association was found between rs3102735 and BMD. **Conclusions**: The rs2073618 polymorphism of the TNFRSF11B gene was associated with BMD Z-scores in survivors of childhood ALL, whereas rs3102735 showed no significant relationship. These findings suggest that genetic variations within the OPG gene may contribute to long-term skeletal health after childhood ALL treatment, but warrant confirmation in larger multicenter studies.

## 1. Introduction

Acute lymphoblastic leukemia (ALL), the most common malignancy in childhood, currently has a five-year overall survival rate exceeding 90% [1,2]. With improving survival rates, there is a growing population of survivors facing numerous long-term treatment-related side effects [3]. The burden of late effects among childhood cancer survivors (CCS) depends on cancer type, treatment intensity, and individual patient characteristics [4]. In ALL survivors, in addition to cardiovascular complications, metabolic syndrome, and endocrine disturbances, reduced bone mineral density (BMD) and increased fracture susceptibility represent particularly concerning health issues [5]. Previous studies have demonstrated that approximately 30% of survivors exhibited reduced BMD 10 years after diagnosis [6].

The Children’s Oncology Group Long-Term Follow-Up (COG-LTFU) Guidelines established a framework for lifelong CCS surveillance. According to these recommendations, BMD assessment should be performed 2 to 5 years after completion of therapy [7]. If initial findings are normal, the patient should be reassessed at 25 years of age, when peak bone mass is expected to be achieved [8]. Dual-energy X-ray absorptiometry (DXA) is considered the standard method for BMD evaluation. In children and adolescents, results must be adjusted for sex, age, and height and expressed as Z-scores [9]. According to the International Society for Clinical Densitometry recommendations, the diagnosis of osteoporosis in this age group relies on fracture history in conjunction with low BMD (Z-score ≤ −2) [10]. However, a Z-score ≤ −1 is considered an indicator of low BMD in CCS [8].

Bone metabolism in ALL patients is impaired as a consequence of anticancer therapy, reduced physical activity, nutritional deficiencies, and/or hormonal disturbances [11]. ALL treatment protocols include agents known to adversely affect bone tissue, particularly corticosteroids and antimetabolites such as methotrexate [12,13]. In addition, evidence suggests that malignant cells may directly influence bone remodeling and density [14,15]. Under these conditions, increased osteoclastic bone resorption together with decreased osteoblastic proliferation and activity results in impaired bone microarchitecture and reduced BMD [16,17]. As a consequence, childhood ALL treatment may interfere with peak bone mass attainment, a major determinant of lifelong bone health [18]. Therefore, many survivors enter adulthood with compromised skeletal integrity, predisposing them to osteoporosis and increased risk of fractures later in life. Understanding the genetic determinants that modulate individual susceptibility to these skeletal complications is essential for personalized risk assessment and targeted preventive strategies.

Physiological regulation of BMD largely depends on the triad comprising receptor activator of nuclear factor kappa B ligand (RANKL), its cognate receptor RANK, and the decoy receptor osteoprotegerin (OPG) [19,20,21]. This signaling system plays a central role in osteoclast formation, activation, and survival, thereby influencing overall bone remodeling and BMD. Binding of RANKL, expressed on osteoblast surfaces, to RANK receptors on osteoclast precursors promotes osteoclast differentiation, enhances bone resorption, and prolongs osteoclast survival. In contrast, OPG acts as a natural antagonist of RANKL and inhibits osteoclastogenesis [22,23].

Genetic variations in the TNFRSF11B gene, which encodes OPG, may modify its expression or biological function. Given OPG’s protective role in bone remodeling, such alterations may contribute to impaired bone metabolism and the development of disorders such as osteoporosis. Previous studies have suggested an association between several OPG single-nucleotide polymorphisms (SNPs), including rs3102735 (A163G) and rs2073618 (G1181C), and susceptibility to osteoporosis. The SNP rs2073618 is located in exon 1 of TNFRSF11B and results in a lysine-to-asparagine amino acid substitution, while rs3102735 is located in the promoter region of the same gene [24,25]. Most previous studies were conducted in adults, and reported findings remain inconsistent [26,27,28]. However, there are no data about the presence of these SNPs and their influence on BMD in CCS.

The present study aimed to investigate the potential influence of rs2073618 and rs3102735 polymorphisms on BMD in survivors of childhood ALL. Given OPG’s role in bone homeostasis and evidence linking TNFRSF11B polymorphisms to BMD variability, this study specifically investigated these two variants as a focused candidate-gene analysis to assess OPG genetic variability’s potential contribution to long-term bone health in survivors of childhood acute lymphoblastic leukemia [29,30]. Although RANK and RANKL are also essential components of this signaling pathway, the investigation of their genetic variants was beyond the scope of the present study.

## 2. Materials and Methods

This single-center cohort study included a retrospective analysis of clinical and treatment-related data, post-treatment DXA results, and a cross-sectional assessment of OPG gene polymorphisms. The local Ethics Committee approved the study (protocol number 28355/15). Written informed consent was obtained from the parents of all participants and from patients older than 15 years.

### 2.1. Patient Population

The study included a total of 60 children diagnosed with ALL between 2005 and 2023 and treated at the Clinic for Pediatrics, University Clinical Center, Niš, Serbia. Eligible participants had completed treatment according to the ALL-IC BFM 2002 or ALL-IC BFM 2009 protocol, and had available medical records. Patients were excluded if they had: (a) infant ALL (disease onset during the first year of life), (b) relapsed disease, or (c) undergone bone marrow transplantation.

During treatment, patients were stratified into risk groups according to the ALL IC-BFM protocols. Based on age at diagnosis, initial white blood cell count, genetic characteristics, and early treatment response, patients were classified as standard-risk (SR), intermediate-risk (IR), or high-risk (HR). The treatment protocol consisted of induction, consolidation, re-induction, and maintenance phases. All the patients received prednisolone during the induction phase (cumulative dose of 1680 mg/m^2^). During re-induction, patients received a low cumulative dose of dexamethasone (250 mg/m^2^), whereas those in the HR group received additional dexamethasone during consolidation, resulting in a higher cumulative dexamethasone exposure (850 mg/m^2^). Methotrexate exposure during consolidation was also recorded: low dose (8000 mg/m^2^) in all SR patients and IR patients with B-cell ALL, and high dose (20,000 mg/m^2^) in HR patients and IR patients with T-cell ALL. Prophylactic cranial irradiation (12 Gy) was administered to patients classified as HR, or IR with T-cell ALL.

BMD was assessed during the second or third year after completion of therapy using DXA on a Hologic Discovery densitometer (Hologic Inc., Bedford, MA, USA). Lumbar spine (L1-L4) areal BMD was measured and expressed as age-, sex-, and height-adjusted Z-scores. Z-score adjustment was performed using the pediatric bone density calculator recommended in the COG-LTFU Guidelines (available at https://zscore.research.chop.edu/calcpedbonedens.php, accessed on 14 April 2026) [7]. A BMD Z-score ≤ −1 was considered indicative of low BMD, consistent with previous studies evaluating skeletal health in CCS [8].

### 2.2. Genetic Analysis

Genomic DNA was isolated from peripheral whole blood samples using a commercially available kit (Invitrogen PureLink Genomic DNA Mini Kit, Thermo Fisher Scientific, Waltham, MA, USA), according to the manufacturer’s instructions. SNPs within the OPG gene (rs2073618 and rs3102735) were genotyped by real-time polymerase chain reaction (PCR) using allele-specific TaqMan SNP Genotyping Assays (Thermo Fisher Scientific, Waltham, MA, USA) on an Applied Biosystems™ 7500 Fast Real-Time PCR System (Foster City, CA, USA). Each PCR was carried out in a final volume of 10.0 μL, containing 5.0 μL of 2 × TaqMan Universal Master Mix (Applied Biosystems, Foster City, CA, USA), 0.25 μL of 40 × TaqMan SNP Genotyping Assay (Assay ID: C_1971047_40 for rs2073618 and C_1971046_10 for rs3102735), 2.0 μL of genomic DNA, and nuclease-free water added to reach the final reaction volume. Thermal cycling conditions were as follows: an initial step at 60 °C for 1 min, enzyme activation at 95 °C for 10 min, followed by 40 cycles of denaturation at 95 °C for 15 s and annealing/extension at 60 °C for 1 min, with a final hold at 60 °C for 1 min. Genotype discrimination and data analysis were performed using the 7500 Software v2.0.6 (Applied Biosystems, Foster City, CA, USA). All analyses were conducted in the Laboratory for Medical Genetics, Faculty of Medicine, University of Niš, Serbia.

### 2.3. Statistical Analysis

Statistical analysis was performed in the R software package (version 4.5.1; R Foundation for Statistical Computing, Vienna, Austria) [31]. Continuous variables were expressed as mean ± standard deviation (SD), while categorical variables were presented as absolute numbers and percentages. The normality of distribution was assessed using the Shapiro–Wilk test. Differences between two independent groups were analyzed using the Mann–Whitney U test for non-normally distributed variables. Differences in categorical variables were tested using the χ^2^ test or Fisher’s exact test, as appropriate. The χ^2^ test was also used to compare observed and expected genotype frequencies and to assess deviation from the Hardy–Weinberg equilibrium.

Differences in mean BMD Z-scores among genotype groups were tested using one-way analysis of variance (ANOVA), followed by Tukey’s post hoc test for pairwise comparisons. Homogeneity of variances was assessed using Levene’s test.

Univariate logistic regression analysis was used to assess the association between demographic, clinical, and genetic variables and reduced BMD. Multivariable logistic regression was subsequently performed to adjust for potential confounders, age, sex, and previous radiotherapy. These covariates were selected a priori based on their established or biologically plausible influence on bone mineral accrual and skeletal health in childhood ALL survivors. The number of covariates was deliberately restricted to minimize the risk of overfitting given the relatively small sample size. Multivariable linear regression analysis was used to identify independent predictors of BMD Z-score. Odds ratios (ORs), regression coefficients (B), standardized coefficients (β), 95% confidence intervals (CIs), and adjusted R^2^ values were reported, as appropriate.

A sensitivity power analysis was performed using G*Power (version 3.1), with α = 0.05 and 80% power, to determine the minimum detectable effect size for the available sample size.

Statistical significance was defined as a *p*-value < 0.05.

## 3. Results

A total of 60 children were included in the study: 35 (58.3%) males and 25 (41.7%) females. Age at ALL diagnosis ranged from 1.2 to 16.0 years, with a mean of 5.83 ± 4.01 years. B-cell ALL was diagnosed in 52 patients (86.7%), whereas T-cell ALL was identified in 8 patients (13.3%). Most patients belonged to the IR group (71.7%) according to the treatment protocol. Prophylactic cranial radiotherapy was performed in 17 patients (28.3%).

The interval between therapy completion and BMD assessment ranged from 1.17 to 2.33 years. Z-score for the total number of patients was −0.564 ± 0.135. Normal BMD, with an average Z-score of −0.005 ± 0.145, was observed in 38 (63.3%) patients, while low BMD, with an average Z-score of −1.528 ± 0.080, was detected in 22 (36.7%) patients. Two patients (3.33%) had Z-scores below −2; however, they did not meet the other criteria required for the diagnosis of osteoporosis. Demographic and clinical characteristics of patients are presented in Table 1.

T-cell ALL was more frequent among patients with low BMD than among those with normal BMD (22.7% vs. 7.9%), and cranial radiotherapy was administered more frequently in the low-BMD group than in the normal-BMD group (40.9% vs. 21.1%). However, the difference did not reach statistical significance for either variable. Overall, we found no significant differences in demographic characteristics, disease-related variables, or treatment exposures between patients with low and normal BMD.

Genotype distributions of rs2073618 and rs3102735 SNPs conformed to the Hardy–Weinberg equilibrium (*p* = 0.053 for rs2073618 and *p* = 0.481 for rs3102735). For rs2073618, the heterozygous CG genotype was most common (61.7%), whereas AA was the predominant genotype at rs3102735 (83.3%) (Table 2). The G allele was more frequent than the C allele at rs2073618 (55.8% vs. 44.2%), while the A allele predominated at rs3102735, accounting for 91.7% of all alleles.

For the rs2073618 SNP, the G allele was more frequent in the normal BMD group (57.9% vs. 52.3%), whereas the C allele was more frequent in the low BMD group (47.7% vs. 42.1%). However, neither allele was significantly associated with reduced BMD (OR = 0.80, 95% CI: 0.38–1.67; *p* = 0.550). No significant difference was observed in allele frequencies for the rs3102735 SNP.

A sensitivity power analysis was performed for BMD analyzed as a binary outcome correlating with the rs2073618 SNP. With a total sample size of 60 participants, a significance level of 0.05, and 80% power, the study could detect an effect size of approximately Cramér’s V = 0.40 for a 2 × 3 comparison of BMD status and genotype.

Univariate logistic regression analyses showed that none of the evaluated clinical or genetic variables was significantly associated with reduced BMD (Table 3).

After adjustment for age, sex, and radiotherapy, we found no association between the rs2073618 SNP and reduced BMD (Table 4).

**Figure 1:**
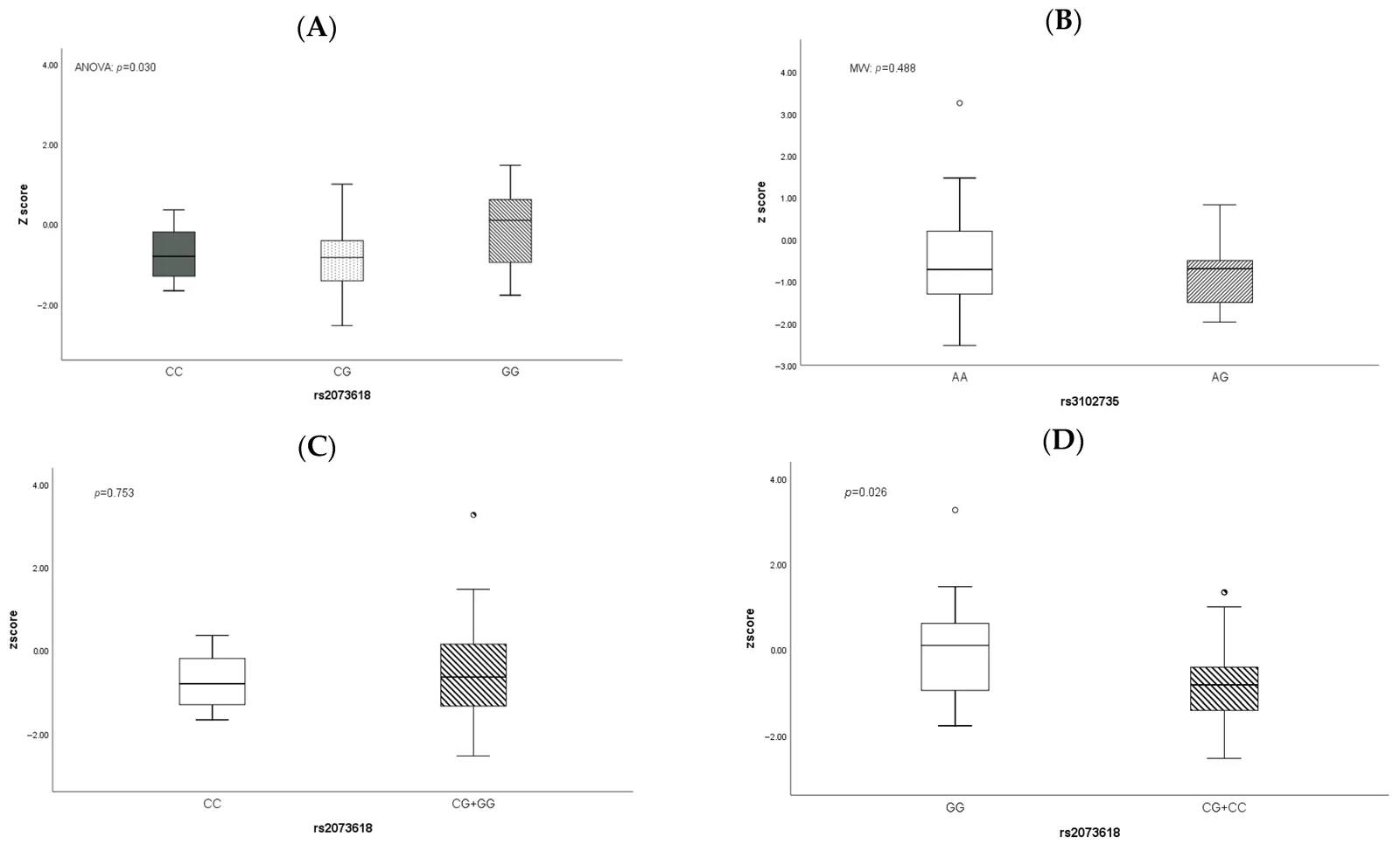
Distribution of BMD Z-scores across OPG rs2073618 and rs3102735 genotype groups. (**A**) Comparison of BMD Z-scores between rs2073618 genotype groups (CC, CG, GG); (**B**) Comparison of BMD Z-scores between rs3102735 genotype groups (AA, AG); (**C**) Comparison of BMD Z-scores between rs2073618 genotype groups (CC vs. CG + GG); (**D**) Comparison of BMD Z-scores between rs2073618 genotype groups (GG vs. CG + CC).

Mean BMD Z-scores differed significantly among rs2073618 genotype groups (one-way ANOVA, F = 3.713, *p* = 0.030). Carriers of the CG genotype exhibited the lowest Z-score values (−0.78 ± 0.92), followed by CC carriers (−0.74 ± 0.70), whereas the highest values were observed among GG carriers (0.05 ± 1.29) (Figure 1A).

Tukey’s post hoc analysis demonstrated significantly higher BMD Z-scores in GG carriers than in CG carriers (mean difference = 0.821, *p* = 0.026). No significant differences were observed between the CG and CC genotypes (*p* = 0.995) or between the CC and GG genotypes (*p* = 0.185). Patients with the CC genotype had Z-scores comparable to those of CG/GG genotype carriers (−0.73 ± 0.70 vs. −0.54 ± 1.09; Mann–Whitney U = 193.5, *p* = 0.753) (Figure 1C). Conversely, GG carriers had significantly higher Z-scores than carriers of the CC/CG genotypes (−0.05 ± 1.29 vs. −0.78 ± 0.88; Mann–Whitney U = 207.5, *p* = 0.026) (Figure 1D).

No significant difference in BMD Z-score was observed between rs3102735 genotypes (Mann–Whitney U = 215.0, *p* = 0.488). BMD Z-scores were comparable between AA and AG genotype carriers, while individuals carrying the GG genotype were not identified (Figure 1B).

To assess whether the association between rs2073618 genotype and BMD Z-score remained independent of potential confounders, we performed a multivariable linear regression analysis. After adjustment for sex, ALL risk group, cumulative dexamethasone exposure, and age at diagnosis, patients carrying the GG genotype had significantly higher BMD Z-scores than carriers of the CC or CG genotypes (B = 0.404, 95% CI 0.070–0.738, standardized β = 0.337, *p* = 0.019). No other variable remained a significant predictor of BMD Z-score (Table 5). Although statistically significant, the final analysis explained only 4.2% of the variability in BMD Z-score (adjusted R^2^ = 0.042).

Overall, carriers of the rs2073618 GG genotype had significantly higher BMD Z-scores than carriers of the CC or CG genotypes, whereas rs3102735 showed no statistically significant effect in the study cohort.

## 4. Discussion

In this study, low BMD was identified in 36.7% of childhood ALL survivors, confirming that impaired bone health remains a frequent long-term complication in this population. Although only two patients had Z-scores below −2 and none fulfilled the ISCD criteria for pediatric osteoporosis, the relatively high prevalence of low BMD emphasizes the importance of long-term skeletal surveillance in this population.

The prevalence of low BMD as a complication of early cancer treatment reported in previous studies varies considerably, ranging from 19.3% to 63.3% [6,32,33,34,35,36,37]. Such variability may reflect differences in patient characteristics, treatment protocols, follow-up duration, or age at BMD assessment. Our findings support the concept that skeletal impairment persists in a substantial proportion of survivors despite successful leukemia treatment [32,37,38].

Conclusions about the impact of clinical and treatment characteristics on BMD remain inconsistent across studies. Some authors have reported age as a risk factor for low BMD in ALL survivors [6,34,39,40], or therapy elements such as corticosteroid dose [6] or methotrexate use [17]. In contrast, others found no significant association between treatment intensity and BMD reduction [39]. In our study, we found no significant associations between BMD and demographic, clinical, or treatment-related variables. Although T-cell ALL and cranial radiotherapy were more frequent among patients with low BMD, these differences did not reach statistical significance. These findings suggest that additional biological and genetic determinants may account for variability in BMD among ALL survivors.

The most important finding of this study was the association between the rs2073618 polymorphism and BMD Z-score. Although the G allele was more frequent in participants with normal BMD and the C allele was more frequent in those with low BMD, these findings were not statistically significant and do not support an independent association between this polymorphism and reduced BMD in ALL survivors. While genotype frequencies did not differ between patients with low and normal BMD, individuals carrying the GG genotype had significantly higher BMD Z-scores than carriers of the CG genotype, and this association remained significant after adjustment for sex, risk group, cumulative dexamethasone exposure, and age at diagnosis. These results suggest that rs2073618 may influence BMD as a continuous quantitative characteristic rather than affecting the presence of low BMD. No significant association was demonstrated when BMD was categorized according to a Z-score threshold of <−1, suggesting that clinical relevance should be interpreted cautiously. This discrepancy may partly reflect the loss of statistical information from dichotomizing a continuous outcome, along with the study’s limited sample size. These findings indicate that rs2073618 may contribute to variability in BMD rather than serving as a marker of clinically relevant low BMD. Although rs2073618 remained independently associated with BMD Z-score, the regression model explained only a small proportion of BMD variability (adjusted R^2^ = 0.042), suggesting that additional genetic, endocrine, nutritional, and lifestyle factors may contribute to skeletal outcomes as a multifactorial trait. Therefore, rs2073618 should be regarded as one of many contributors rather than an isolated determinant of bone health.

The biological plausibility of this association is supported by the central role of the OPG/RANK/RANKL signaling pathway in bone remodeling. The rs2073618 polymorphism has been associated with susceptibility to osteoporosis and bone fractures in several populations, although published findings remain inconsistent [25,28,40,41,42,43]. Differences in ethnicity, age, underlying diseases, environmental influences, and sample size may explain these discrepancies. Most previously published studies were conducted in older individuals and postmenopausal women. Only a limited number of studies have investigated the possible association between OPG polymorphisms and low BMD in children with chronic diseases [42]. Our results support the potential involvement of rs2073618 in bone metabolism; however, the direction of the observed association differs from that reported in some previous studies [28,41].

In contrast, we found no significant association between the rs3102735 polymorphism and BMD in our cohort. The predominance of the AA genotype, the low frequency of the G allele, and the absence of one genotype subgroup may have limited statistical power to detect weaker genetic effects. Other authors have also reported conflicting results regarding the association between rs3102735 and osteoporosis, with differences largely attributed to ethnic variations among study populations [26,28,41,43,44].

Although OPG is an important regulator of osteoclast activity, RANK, RANKL, and OPG interact in a coordinated manner to control bone remodeling. Therefore, the potential contribution of genetic variants in TNFRSF11A (RANK) and TNFSF11 (RANKL), as well as interactions among these genes, could not be evaluated. Future studies involving larger cohorts and comprehensive genetic analysis of the RANK/RANKL/OPG pathway may provide further insight into the genetic determinants of reduced BMD in childhood ALL survivors.

Genetic susceptibility to impaired bone health in childhood ALL survivors is likely to be complex and polygenic, and not restricted to the RANK/RANKL/OPG pathway. Previous studies have reported associations between BMD and genetic variants involved in vitamin D signaling, glucocorticoid response, osteogenesis, and bone matrix formation. In particular, polymorphisms in COL11A1 and NELL1 have been associated with decreases in BMD Z-scores during ALL treatment, while associations with variants in VDR, MTHFR, MTRR, CRHR1, and RAPGEF5 have also been reported [45,46]. More recently, a polygenic score reflecting genetic susceptibility to bone characteristics was associated with BMD at multiple skeletal sites in childhood ALL survivors, supporting the contribution of inherited genetic factors to long-term bone health [47]. In this context, our findings regarding TNFRSF11B rs2073618 add to the evidence that genetic variation in pathways regulating bone remodeling may contribute to interindividual variability in BMD after ALL treatment. However, given the modest sample size and the absence of a significant association when BMD was analyzed as a binary outcome, these findings should be regarded as exploratory and require confirmation in larger cohorts.

The main limitations of this study include the relatively small sample size and single-center design; therefore, observed differences should be interpreted with caution. Nevertheless, genotype distributions for both polymorphisms were in Hardy–Weinberg equilibrium, supporting the reliability of the genetic analyses. Sensitivity analysis indicated that the available sample provided 80% power to detect only moderate-to-large associations (Cramér’s V ≈ 0.40) between genotype and binary BMD status. Therefore, the absence of a significant association with reduced BMD should not be interpreted as evidence of the absence of smaller genetic effects. In addition, factors such as physical activity or vitamin D and calcium intake were not evaluated, although these may substantially influence BMD. Another important aspect to address is evaluating bone turnover markers that could provide further insight into the pathophysiology of osteopenia after treatment for childhood ALL.

Despite these limitations, this study contributes to the limited body of evidence on the genetic determinants of bone health in childhood ALL survivors. Identifying genetic variants associated with reduced BMD could help in early recognition of patients at increased risk for skeletal complications and enable earlier monitoring and preventive interventions.

In conclusion, our study suggests that the rs2073618 polymorphism may be associated with BMD Z-score in pediatric ALL survivors, with a potentially beneficial role of the GG genotype. In contrast, no association was observed for rs3102735. Larger multicenter studies are needed to confirm these observations and clarify the role of OPG genetic variants in long-term skeletal outcomes after ALL treatment.

## Figures and Tables

**Table 1:** Demographic, clinical, and treatment characteristics of patients according to BMD status.

Characteristic	Total		Low BMD (Z ≤ −1) N = 22		Normal BMD (Z > −1) N = 38		*p*-Value
	mean ± SD		mean ± SD		mean ± SD		
Age at diagnosis (years)	5.83 ± 4.01		6.03 ± 3.99		5.71 ± 4.07		0.776 ^1^
BMI at diagnosis (kg/m^2^)	16.18 ± 3.02		16.20 ± 3.61		16.17 ± 2.67		0.667 ^1^
	N	%	N	%	N	%	
Sex							0.365 ^2^
Male	35	58.3	15	68.2	20	52.6	
Female	25	41.7	7	31.8	18	47.7	
Immunophenotype							0.129 ^3^
B-cell	52	86.8	17	77.3	35	92.1	
T-cell	8	13.3	5	22.7	3	7.9	
Risk group							0.983 ^3^
SR	6	10.0	2	9.1	4	10.5	
IR	43	71.7	16	72.7	27	71.1	
HR	11	18.3	4	18.2	7	18.4	
Dexamethasone dose							1.000 ^3^
Low	49	81.7	18	81.8	31	81.6	
High	11	18.3	4	18.2	7	18.4	
MTX dose							0.451 ^2^
Low	43	71.7	14	63.6	29	76.3	
High	17	28.3	8	36.4	9	23.7	
Radiotherapy	17	28.3	9	40.9	8	21.1	0.178 ^2^
Long bone fractures after treatment	4	6.67	2	9.10	2	5.30	0.619 ^3^

^1^ Mann–Whitney test, ^2^ χ^2^-test, ^3^ Fisher’s exact test, SR—standard risk, IR—intermediate risk, HR—high risk, MTX—methotrexate.

**Table 2:** Distribution of rs2073618 and rs3102735 genotypes and allele frequencies according to BMD status in childhood ALL survivors.

Characteristic	Total		Low BMD (Z ≤ −1) N = 22		Normal BMD (Z > −1) N = 38		*p*-Value
	N	%	N	%	N	%	
rs2073618							0.633 ^1^
CC	8	13.3	3	13.6	5	13.2	
CG	37	61.7	15	68.2	22	57.9	
GG	15	25.0	4	18.2	11	28.9	
Allele							0.550 ^1^
C	53	44.2	21	47.7	32	42.1	
G	67	55.8	23	52.3	44	57.9	
rs3102735							1.000 ^1^
AA	50	83.3	18	81.8	32	84.2	
AG	10	16.7	4	18.2	6	15.8	
Allele							1.000 ^2^
A	110	91.7	40	90.9	70	92.1	
G	10	8.3	4	9.1	6	7.9	

^1^ χ^2^-test, ^2^ Fisher’s exact tests.

**Table 3:** Univariate logistic regression analysis of factors associated with reduced BMD.

Variable	Comparison	OR (95% CI)	*p*-Value
Age	per year	1.020 (0.895–1.163)	0.763
Sex	female vs. male	0.519 (0.173–1.558)	0.242
Immunophenotype	B-cell vs. T-cell	0.291 (0.062–1.365)	0.118
MTX dose	low vs. high	0.543 (0.173–1.709)	0.297
Dexamethasone dose	low vs. high	0.699 (0.161–3.035)	0.633
Radiotherapy	yes vs. no	2.596 (0.819–8.228)	0.105
rs2073618	CC vs. (CG + GG)	1.042 (0.224–4.854)	0.958
rs2073618	GG vs. (CG + CC)	0.545 (0.150–1.982)	0.357
rs3102735	AA vs. AG	0.844 (0.210–3.390)	0.811

OR—odds ratio, CI—confidence interval.

**Table 4:** Multivariable logistic regression analysis of factors associated with reduced BMD.

Variable	Adjusted OR (95%CI)	*p*-Value	Adjusted OR (95% CI)	*p*-Value
	GG vs. (CG + CC)		CC vs. (CG + GG)	
Age	1.029 (0.896–1.182)	0.685	1.016 (0.887–1.164)	0.818
Sex	0.629 (0.194–2.033)	0.438	0.642 (0.199–2.071)	0.458
Radiotherapy	2.240 (0.665–7.550)	0.193	2.265 (0.675–7.601)	0.186
rs2073618	0.504 (0.130–1.958)	0.322	0.877 (0.177–4.360)	0.873

OR—odds ratio, CI—confidence interval.

**Table 5:** Multivariable linear regression analysis of demographic, clinical, and genetic factors associated with BMD Z-score.

	Unstandardized Coefficient		95% CI for B		Standardized Coefficient	*p*-Value
	B	SE			Β	
Constant	−1.469	0.727	−2.926	−0.012		0.048
Sex	0.140	0.277	−0.416	0.696	0.067	0.616
Risk group	0.070	0.254	−0.440	0.580	0.045	0.783
rs2073618 (GG vs. CC + CG)	0.404	0.167	0.070	0.738	0.337	0.019
Dexamethasone exposure	0.054	0.392	−0.732	0.840	0.020	0.891
Age at diagnosis	−0.010	0.036	−0.082	0.061	−0.040	0.774
Dependent variable: BMD Z-score; adjusted R^2^ = 0.042						

SE—standard error; CI—confidence interval; risk group—standard, intermediate, and high risk according to ALL-IC BFM protocols.

## Data Availability

The data presented in this study are available on request from the corresponding author due to privacy reasons.

## Abbreviations

The following abbreviations are used in this manuscript:

ALL	Acute lymphoblastic leukemia
CCS	Childhood cancer survivors
BMD	Bone mineral density
COG-LTFU	Children’s Oncology Group Long-Term Follow-Up
DXA	Dual-energy X-ray absorptiometry
RANK	Receptor activator of nuclear factor kappa
RANKL	Receptor activator of nuclear factor kappa B ligand
OPG	Osteoprotegerin
ALL IC BFM	Acute lymphoblastic leukemia international cooperative Berlin-Frankfurt-Münster
SNP	Single-nucleotide polymorphism
SR	Standard risk
IR	Intermediate risk
HR	High risk
